# A Truncated AdeS Kinase Protein Generated by IS*Aba1* Insertion Correlates with Tigecycline Resistance in *Acinetobacter baumannii*

**DOI:** 10.1371/journal.pone.0049534

**Published:** 2012-11-14

**Authors:** Jun-Ren Sun, Cherng-Lih Perng, Ming-Chin Chan, Yuji Morita, Jung-Chung Lin, Chih-Mao Su, Wei-Yao Wang, Tein-Yao Chang, Tzong-Shi Chiueh

**Affiliations:** 1 Graduate Institute of Medical Science, National Defense Medical Center, Taipei, Taiwan; 2 Division of Clinical Pathology, Department of Pathology, Tri-Service General Hospital, Taipei, Taiwan; 3 Infection Control Office, Tri-Service General Hospital, Taipei, Taiwan; 4 Graduate Institute of Pathology, National Defense Medical Center, Taipei, Taiwan; 5 Department of Microbiology, School of Pharmacy, Aichi Gakuin University, Nagoya, Aichi, Japan; 6 Division of Infectious Diseases and Tropical Medicine, Department of Medicine, Tri-Service-General Hospital, Taipei, Taiwan; 7 Department of Microbiology and Immunology, National Defense Medical Center, Taipei, Taiwan; 8 Division of Infectious Disease, Fong-Yuan Hospital, Taichung, Taiwan; University of Birmingham, United Kingdom

## Abstract

Over-expression of AdeABC efflux pump stimulated continuously by the mutated AdeRS two component system has been found to result in antimicrobial resistance, even tigecycline (TGC) resistance, in multidrug-resistant *Acinetobacter baumannii* (MRAB). Although the insertion sequence, IS*Aba1*, contributes to one of the AdeRS mutations, the detail mechanism remains unclear. In the present study we collected 130 TGC-resistant isolates from 317 carbapenem resistant MRAB (MRAB-C) isolates, and 38 of them were characterized with IS*Aba1* insertion in the *adeS* gene. The relationship between the expression of AdeABC efflux pump and TGC resistant was verified indirectly by successfully reducing TGC resistance with NMP, an efflux pump inhibitor. Further analysis showed that the remaining gene following the IS*Aba1* insertion was still transcribed to generate a truncated AdeS protein by the *P*out promoter on IS*Aba1* instead of frame shift or pre-termination. Through introducing a series of recombinant *adeRS* constructs into a *adeRS* knockout strain, we demonstrated the truncated AdeS protein was constitutively produced and stimulating the expression of AdeABC efflux pump via interaction with AdeR. Our findings suggest a mechanism of antimicrobial resistance induced by an aberrant cytoplasmic sensor derived from an insertion element.

## Introduction

*Acinetobacter baumannii*, a Gram-negative coccobacillus usually found in soil and water, has emerged as a highly problematic nosocomial pathogen [1]. *A. baumannii* constitutes a major public health problem due to its intrinsic resilience to numerous drugs and its ability to readily acquire new resistance determinants [2], [3]. Multidrug-resistant *A. baumannii* resistant to carbapenem (MRAB-C) has been increasingly reported worldwide, which raises serious concern about the limited antimicrobial treatment options [4].

Multidrug efflux pumps belonging to the resistance-nodulation-cell division (RND) family have been shown to play an important role in the antimicrobial resistance of *A. baumannii* [3], [5]. To date, three different RND pumps have been identified in *A. baumannii*, namely AdeABC, AdeIJK and AdeFGH. AdeABC efflux pump is the first characterized RND system in *A. baumannii* [3]. Overexpression of the AdeABC efflux pump has been shown to pump out aminoglycosides, beta-lactam, fluoroquinolone, tetracycline, macrolide, chloramphenicol, trimethoprim, and even tigecycline (TGC). TGC is the first member of the glycylcycline and is considered a bacteriostatic drug with high efficacy in treating a broad range of antibiotic-resistant bacteria [4], [6]. Previous studies have shown that TGC has poor activities against MRAB-C due to over-expression of the AdeABC efflux pump system [3], [7], [8], [9], [10], [11]. The AdeIJK efflux pump contributes minimally to acquired resistance due to its toxicity for the host [12]. The third efflux pump, AdeFGH, its overexpression confers high-level resistance to most antibiotics except β-lactam and aminoglycosides [13]. Its overexpression is therefore least expected to result in the multidrug resistance in MRAB-C isolates. The expression of AdeABC efflux pump is tightly regulated by the two-component system which contains a sensor kinase (SK) AdeS and a response regulator (RR) AdeR, encoded by the *adeRS* operon. The *adeRS* operon is located at the upstream of *adeABC* operon and is transcribed in the opposite direction [3], [7], [14]. The promoters of the *adeABC* operon and the *adeRS* operon were predicted at the region between the two operons [14].

In micro-organisms, the two-component system is a very important signal transduction system for adaptation to drastic and immediate changes in external or internal environmental conditions [15]. This system is usually composed of a RR and a SK. The SK monitor's certain signal is caused by environmental changes. Signal bound to sensor domains of SK normally results in inducting an ATP dependent auto-phosphorylation of the histidine kinase domain in the catalytic core of the enzyme and transmitting the information to RR via a phosphoryl-transfer reaction to aspartate residue in RR. The RR is shifted to its active state by the phosphate group received and is directly bound to the promoter region of its target genes as a transcriptional regulator [16]. The typical type of SK is a homodimeric integral membrane protein in which the sensor domain, direct detection of extracellular signals, is formed by an extracellular loop contained between two membrane-spanning segments. The transmitter domain contains a histidine kinase A (HisKA) domain, and an ATP-binding domain follows the last transmembrane segment at the C-terminus and is localized within the cytoplasm [15]. However, some SKs which deviate from the typical type of SK model with their sensor domains within the membrane or fully cytoplasmic are found in bacteria, fungi, and plant [16]. The cytoplasmic SK is a protein located in the cytoplasm comprising a sensor domain and transmitter domain, without a transmembrane domain [16], [17], [18], [19], [20].

Several families of Insertion sequence (IS) in *A. baumannii* have been associated with acquired antimicrobial resistance mechanisms [21]. Many IS elements are related to antibiotic resistance through two mechanisms. Some of the IS elements contain promoters which can enhance the expression of their downstream antibiotic resistance genes [22]. IS*Aba1* has been found in upstream of *ampC*, *bla*_OXA-23_, *bla*_OXA-27_, *bla*_OXA-51_ and *sul*II antibiotic resistance genes [22], [23], [24], [25], [26], [27], [28], [29], [30]. Other elements, such as IS*Aba2*, IS*Aba3*, and IS*Aba4* contain preceding promoters that enhance the expression of carbapenem resistant genes, *bla*_OXA-58_ and the *bla*_OXA-23_ genes [31], [32], [33]. Moreover, the IS element may be inserted into and may disrupt the coding region of an outer membrane protein gene. The loss of the outer membrane protein results in resistance by blocking the input of antibiotics. Previous research described that an insertion mutation within the *carO* outer membrane protein gene by the IS*Aba1*, IS*Aba125*, or IS*Aba825* element was associated with carbapenem resistant [22], [30], [34].

Previous studies found that IS*Aba1* insertion mutation within the *adeS* gene can enhance overexpression of the AdeABC efflux pump system and cause TGC resistant [9]. Excessive transcription of *adeABC* operon was triggered due to a strong promoter in IS*Aba1* or the disruption of the AdeS protein. However, the detail mechanism regarding how the IS*Aba1* element controls the AdeABC efflux pump over- expression remains unknown. In the present study, we investigated the prevalence, genotype, AdeABC efflux pump expression and TGC resistance pattern of MRAB-C clinical isolates in which *adeRS* operon was uniquely destroyed by insertion of an IS element. Further, we identified a truncated cytoplasmic AdeS encoded by an extra operon driven by the promoter within the IS*Aba1* element. The truncated AdeS protein was then responsible for enhancing AdeABC efflux pump over- expression.

## Materials and Methods

### Bacterial strains, growth media and plasmids

A total of 317 distinct clinical isolates of MRAB-C were collected in this study at Tri-Service General Hospital from 2008 to 2010. The bacterial strains and plasmids used in this study are listed in Table 1. Bacteria were grown routinely in Luria–Bertani (LB) broth (BD Difco, Franklin Lakes, NJ) at 37°C with shaking. Plasmids were maintained and selected in *E. coli* and *A. baumannii* hosts using a medium supplemented with the following antibiotics: ampicillin (Sigma-Aldrich, Poole, UK) (100 mg/L); kanamycin (Sigma-Aldrich) (50 mg/L); and tetracycline (Sigma-Aldrich) (5 mg/L).

**Table 1:** Bacterial strains and plasmids used in this study.

Strains or plasmids		Relevant characteristics	Reference
*E. coli* strains	DH5α	Φ80d, *lacZM15*, *endA1*, *recA1*,*hsdR17* (r−, m+), supE44, thi-1, gyrA96, relA1, F– (lacZYA- argF)U169	Lab stock
*Acinetobacter baumannii* strains	ATCC 15151	*A. baumannii* reference strain	ATCC
	ABmut02	Derived from BCRC15884. *adeRS* mutant obtained by plasmid insertion. KanR, AmpR	This study
	AB039	clinical MDR strain, TGC MIC = 2 mg/L, *adeRS* without IS element insertion	
	AB090	clinical MDR strain, TGC MIC = 1 mg/L, *adeS* mutant obtained by IS*aba*1 insertion	This study
	AB096	clinical MDR strain, TGC MIC = 16 mg/L, *adeRS* without IS element insertion	
	AB260	clinical MDR strain, TGC MIC = 1 mg/L, *adeS* mutant obtained by IS*aba*1 insertion	This study
	AB293	clinical MDR strain, TGC MIC = 16 mg/L, *adeS* mutant obtained by IS*aba*1 insertion	This study
	AB392	clinical MDR strain, TGC MIC≦ 0.5 mg/L, *adeR* mutant obtained by IS*aba125* insertion	This study
Plasmids	pMU125	*E. coli-Acinetobacter* shuttle plasmid with A. baumannii replication origin	[37]
	pCR2.1-Topo	Suicide plasmid for *A. baumannii*. KanR, AmpR	Invitrogen
	pCR2.1-*adeR*int	pCR2.1-Topo containing a 513-bp internal fragment of the adeR. KanR, AmpR	This study
	pYM102	mini-CTX::*lacI^q^*-P_T7_-*lacZ*	[40]
	pS01	*E. coli-Acinetobacter* shuttle plasmid with *A. baumannii* replication origin; mini-CTX::lacIq-PT7-lacZ	This study
	pRS-*adeRS* (wt)	Derived from pRS, mini-CTX:: *adeRS*-P*ade*-*lacZ* (*adeRS* from ATCC 15151)	This study
	pRS-*adeRS* (AB090)	Derived from pRS, mini-CTX:: *adeRS*-P*ade*-*lacZ* (adeRS from AB260)	This study
	pRS-*adeRS* (AB293)	Derived from pRS, mini-CTX:: *adeRS*-P*ade*-*lacZ* (*adeRS* from AB293)	This study
	pRS-*adeR* (wt)	Derived from pRS, mini-CTX:: *adeR*-P*ade*-*lacZ* (*adeR* from ATCC 15151)	This study
	pRS-*adeR* (AB260)	Derived from pRS, mini-CTX:: *adeR*-P*ade*-*lacZ* (*adeR* from AB260)	This study
	pRS-*adeR* (AB293)	Derived from pRS, mini-CTX:: *adeR*-P*ade*-*lacZ* (*adeR* from AB293)	This study
	pRS-P*ade*-Pout-truncated *adeS*	Derived from pRS-adeRS (AB293), *Sph*I digested and self-ligatioin. mini-CTX:: truncated *adeS*-P*out*-Pade-*lacZ*	This study
	pRS-P*out*-truncated *adeS*	Derived from pRS, mini-CTX:: truncated *adeS* -Pout-*lacZ* (soluble adeS from AB293)	This study
	pRS-P*out*-truncated *adeS* (SDM)	Derived from pRS_P*out*_truncated *adeS*, mutated at the start code ATG to AAG by site-directed mutagenesis	This study

### Antimicrobial susceptibility tests

All bacterial cultures were prepared on the day of antimicrobial susceptibility testing. The U.S. Food and Drug Administration (FDA) recommended tigecycline (TGC) susceptibility breakpoints for *Enterobacteriaceae* (susceptible MIC, ≦2 g/L; intermediate MIC, >2 and <8 g/L; resistant MIC, ≧8 g/L) were used as MIC interpretation criteria. TGC (Wyeth, Madison, NJ) powder was obtained from commercial sources and was prepared in a solution with sterilized water, and the solution was then frozen in aliquots at −80°C. TGC MICs were initially determined by the E-test (AB Biodisk, Solna, Sweden) and confirmed by the agar dilution test using Mueller-Hinton II agar (BD Difco). Susceptibility tests of other antimicrobials were performed by the VITEK-2 system (bioMérieux, Marcy l'Etoile, France). All strains were tested for susceptibility to the following antibiotics: ampicillin, cefazolin, gentamicin, amikacin, ampicillin/sulbactam, piperacillin/tazobactam, trimethoprim/sulfamethoxazole, ceftazidime, ceftriaxone, imipenem, ciprofloxacin, cefepime, nitrofurantoin, tigecycline and levofloxacin. To verify the over- expression of efflux pump, we prepared Mueller–Hinton (M-H) agar plates with and without adding the efflux pump inhibitor 1-(1-naphthylmethyl)-piperazine (NMP) (Sigma-Aldrich). The final concentration of NMP in the M-H agar was 64 mg/L. The MICs of TGC in the presence or absence of NMP were determined by the E-test described above.

### DNA manipulations

Genomic DNA was isolated using the protocol suggested in the literature [35]. Plasmid DNA was isolated by the Viogene plasmid DNA extraction miniprep system (Viogene biotek corporation, Taiwan). DNA concentration measurements were performed with a NanoDrop ND-1000 spectrophotometer (NanoDrop Technologies, Inc., Wilmington, DE, USA). Standard PCR amplifications were performed with the DyNAzyme DNA polymerase (Finnzymes, Espoo, Finland). When necessary, high fidelity and blunt-ended PCR products were amplified with Phusion DNA Polymerase (Finnzymes). PCR products were purified with Virogene Gel and PCR Clean-Up kit (Viogene). Oligonucleotide was synthesized by Genomics BioScience and Technologies, Inc. (Taiwan). All oligonucleotides used in the study are listed in Table 2. Thermal cycle reactions were performed using the 9700 GeneAmp thermocycler (Applied Biosystems, Foster City, CA). Nucleotide sequencing was performed with BigDye Terminator Cycle Sequencing kit (Applied Biosystems) according to the manufacturer's instructions. Capillary electrophoresis was performed with a 3730XL Genetic Analyzer (Applied Biosystems) and then analyzed by BioEdit software (http://www.mbio.ncsu.edu/BioEdit/bioedit.html). Restriction enzymes were purchased from New England Biolabs (New England Biolabs, Beverly, MA) and used according to the manufacturer's recommendation. Fast-Link DNA Ligation Kit was purchased from Epicentre (Epicentre, Madison, Wisconsin) and used according to the manufacturer's recommendation. Competent cells were prepared and transformed by electroporation as previously described [36]. The sequence was analyzed with the NCBI BlastX bioinformatic tool (http://blast.ncbi.nlm.nih.gov/Blast.cgi) and the NCBI open reading frame (ORF) finder (http://www.ncbi.nlm.nih.gov/projects/gorf/).

**Table 2:** Oligonucleotides used in this study.

Primer name	Sequence	Features/purpose	Source
adeRS_1f	ATGTTTGATCATTCTTTTTCTTTTG	Detection the IS element insertion within the *adeRS* operon and Sequencing of *adeRS* operon	[41]
adeRS_1849r	TTAGTTATTCATAGAAATTTTTATG		
adeRS_689r	TTAATTAACATTTGAAATATG		
adeRS_776f	ATGAAAAGTAAGTTAGGAATTAGTAAG		
ISAba1-F	CATTGGCATTAAACTGAGGAGAAA	with ade RS_1849r primer to confirme the nsertion of IS*aba1* within the *adeS* operon	This study
ISAba1-R	TTGGAAATGGGGAAAACGAA	with ade RS_1f primer to confirme the insertion of IS*aba1* within the *adeS* operon	This study
pWH1266_ori_PstI_F	GCCTGCAGGATCGTAGAAATATCTATGA	Cloning of *A. baumannii* replication origin from pMU125 with engineered *Pst*I site	This study
pWH1266_ori_PstI_SmaI_R	AACTGCAGACCCGGGGGATTTTAACATTTTGCGTTG		
AdeA_promoter_f_BamHI	CACGGATCCAACCTAGTGAGTTTTTGATGTTCG	Cloning *adeRS* operon and promoter *ade* with engineered SmaI and BamHI site into pRS to form pRS- *adeRS*	This study
AdeS_r_SmaI	CGCCCCGGGTTAGTTATTCATAGAAATTTTTATG		
AdeR_r_SmaI	CACCCCGGGTATTTAGGCGTCATCTTTTACAGC	With AdeA_promoter_f *Bam*HI primer to clone *adeR* operon and promoter ade with engineered *Sma*I and *Bam*HI site into pRS to form pRS- *adeR*	This study
ISaba1Pout_f_BamHI	CACGGATCCAAGCATGATGAGCGCAAAG	With AdeS_r *Sma*I primer to clone soluble *adeS* operon and IS*aba1* P*out* with engineered *Sma*I and *Bam*HI site into pRS to form pRS-P*out*-soluble *adeS*	This study
adeR_6f	TGATCATTCTTTTTCTTTTGATTGCCAAGA	Cloning the partial sequence of *adeR* into pCR2.1- Topo to form pTOPO*adeR*	This study
adeR_514r	CAGTCAGCGTCAGATTAAGCA		
adeA_F	TTGATCGTGCTTCTATTCCTCAAG	RT- PCR of *adeA* gene	
adeA_R	GGCTCGCCACTGATATTACGTT		
adeB_F	GGATTATGGCGACAGAAGGA	RT- PCR of *adeB* gene	
adeB_R	AATACTGCCGCCAATACCAG		
QPCR_adeRS1661_f	ACCGAGTTCCAAGACGAT	RT- PCR of *adeS* gene	This study
QPCR_adeRS1782_r	CCTTTCAGTGCCACAATA		
QPCR_adeR96_f	AAAACGTGAAGGCATGAGTG	RT- PCR of *adeR* gene	This study
QPCR_adeR220_r	CTTCCCAACCGTTTAATTCG		
rpoB_f	TCCGCACGTAAAGTAGGAAC	RT- PCR of *rpoB* gene	
rpoB_r	ATGCCGCCTGAAAAAGTAAC		
TruncatedAdeS_SDMut_sense	ATAATTTTAATGATAAGGCTCAAA**A**GC	change the start code ATG to AAG in soluble *adeS* by site-directed mutagenesis at pRS-Pout-soluble *adeS*	This study

### Quantitative real-time PCR

To detect the expression of *adeR, adeS, adeA and adeB* gene, quantitative real-time RT-PCR was performed individually for each. Primers for the quantitative real-time were designed using Primer3 software. The efficiency of each primer pair in quantitative real-time PCR has been determined by a 10-fold serial dilution of the template. By plotting the log values of template concentrations on the x-axis and the Ct values on the y-axis, standard linear lines with slopes (m) ranging from −3.58 to −3.27 were obtained. We obtained 95.1∼105.0% amplification efficiencies (E_Amp_) for the primer pairs based on the calculation equation, E_Amp_ = 10^−1/m^−1. *A. baumannii* cells were grown aerobically in LB broth until reaching the mid-log phase. DNaseI-treated RNA templates were prepared using an RNeasy kit (QIAGEN Sciences, MD). The concentrations of the RNA were quantified with a spectrophotometer. Reverse transcription was performed using a high-capacity cDNA archive kit (Applied Biosystems) with 200 ng of RNA in a 20 µL reaction mixture containing 1× (each) reverse transcription buffer, deoxynucleoside triphosphate mix, and random primers as well as 2.5 U/ml multiscribe reverse transcriptase. Negative-control reactions included equal concentrations of RNA and all reagents while omitting reverse transcriptase. Incubation for 10 min at 25°C followed by 2 h at 37°C was carried out with a GeneAmp PCR System 9700 thermal cycler (Applied Biosystems). Real time PCR was performed with the DyNAmo™ Flash SYBR Green qPCR Kit (Finnzymes) and a 1∶10 final dilution of the cDNA product on the ABI PRISM 7500 Real-Time PCR system (Applied Biosystems). Each PCR analysis contained one primer pair. Cycling conditions were as follows: denature at 95°C for 10 min, followed with 40 cycles of denature at 95°C for 15 s and annealing/extension at 56°C for 30 s, and finalized with 1 cycle at 95°C for 30 s and 28°C for 30 s. Following PCR cycling, melting point data were collected and the dissociation curve was examined for each well. The critical threshold cycle (Ct) numbers were determined by Sequence Detection Systems version 2.2.2 (Applied Biosystems). In order to omit efficiency variation among primer pairs, ΔCt and ΔΔCt were applied for comparing the gene expression level. The ΔCt for gene expression was calculated against that for the *rpoB* housekeeping gene, and the ΔΔCt was calculated against that for the TGC-susceptible control strain (ATCC 15151, wt) as suggested in the literature.

### Knockout the *adeRS* operon by homologous recombination

Plasmid inserted with the *adeRS* operon of *A. baumannii* was carried out as previously described [36]. Briefly, kanamycin and ampicillin resistant plasmid pCR2.1-Topo, unable to replicate in *A. baumannii*, was used as a suicide vector. An internal fragment (513 bp) of the *adeR* gene was amplified by PCR with AdeR_6f and AdeR_514r primers (Table 2) from a template of *A. baumannii* ATCC 15151 genomic DNA. The PCR product was cloned into the pCR2.1-Topo vector and electroporated into *E. coli* to yield the pCR2.1-*adeR*int plasmid (Table 1). Recombinant plasmid (100 ng) was then introduced in the kanamycin and ampicillin susceptible *A. baumannii* ATCC 15151 by electroporation [36]. Mutants were selected on LB agar plates containing kanamycin and ampicillin. Inactivation of the *adeRS* operon by insertion of the plasmid via a single crossover recombination was confirmed by sequencing the amplified PCR products with the AdeA_promoter_f_*BamH*I and AdeRS_1849r primer pairs (Table 2).

### Construction of the *E. coli*-*Acinetobacter* shuttle vector pS01

The DNA fragment of *A. baumannii* replication origin was obtained by PCR using primers pWH1266_ori_*Pst*I_F and pWH1266_ori_*Pst*I_*Sma*I_R (Table 2) in a reaction mixture (50 µL) containing 1 U of Phusion DNA Polymerase, 0.2 mM of each deoxynucleoside triphosphate, 1× PCR buffer, 5 pmol of each primer, 2 mM MgSO_4_ and 10 ng of pMU125 DNA as the template [37]. For PCR, the reaction mixture was subjected to an initial 1 min denaturation step at 98°C, followed by 35 cycles of 10 s at 98°C, 10 s at 55°C, and 30 s at 72°C and a 10 min final elongation at 72°C. The purified PCR product was then cloned into plasmid pYM102 through the *Pst* I site. The resulting construct, pS01, was sequenced to verify the absence of PCR-introduced mutations.

### Construction of pRS series for expressing the *adeRS*, *adeR* and Pout-truncated *adeS* genes

The plasmid pS01 was digested with *Sma*I and *Bam*HI for 2 h at 37°C (or 25°C in the case of *Sma*I) to delete its *lacI* ^q^ gene and T7 early gene promoter. The digested plasmids were ligated with the respective gene fragments to generate a series of recombinant pRS clones, including pRS-*adeRS* (wt), pRS-*adeRS* (AB090), pRS-*adeRS* (AB293), pRS-*adeR* (wt), pRS-*adeR* (AB260), pRS-*adeR* (AB293) and pRS-*P*out-truncated_*adeS*. The *adeRS* and *adeR* gene fragments of ATCC 15151 and AB260 were amplified by PCR using common forward primer AdeA_promoter_f_*Bam*HI with reverse primers AdeS_r_*Sma*I and AdeR_r_*Sma*I, respectively (Table 2). The Pout-truncated *adeS* gene fragment of AB293 was amplified by PCR using primers IS*Aba1*Pout_f_*BamH*I and AdeS_r_*Sma*I. Another plasmid pRS-Pade-Pout-truncated *adeS* was obtained by splicing out the DNA fragment between *P*ade and *P*out of ISAbaI in pRS-*adeRS* (AB293) with *Sph*I digestion and re-ligation.

### Site Directed Mutagenesis

The site-directed mutagenesis (SDM) protocol was modified from the previous study [38]. We used plasmid pRS-Pout-truncated *adeS* as the template for SDM to disrupt start codon (ATG to AAG). The plasmid was amplified using a mutation bearing primer TruncatedAdeS_SDMut_sense in PCR reaction mixture (25 µL) containing 1 µg template, 1 U of Phusion DNA Polymerase, 0.2 mM of each deoxynucleoside triphosphate, 1× PCR buffer, 5 pmol of SDM primer, 2 mM MgSO_4_ and 10% (vol/vol) dimethyl sulfoxide (DMSO). For PCR, the reaction mixture was subjected to an initial 30 sec denaturation step at 98°C, followed by 28 cycles of 10 s at 98°C, 30 s at 50°C, and 4 min at 72°C and a 10-min final elongation at 72°C. The PCR product was further treated with 5 U of *Dpn*I at 37°C for 1 hr. The *Dpn*I-treated PCR product was directly transformed into DH5α competent cells. Transformants were incubated in 5 ml LB medium containing 5 mg/L tetracycline overnight. Tetracycline resistant colonies were isolated and extracted for plasmid content. Mutant start codon in the successfully transformed plasmid was confirmed by DNA sequencing.

### Pulsed-field gel electrophoresis

All clinical *A. baumannii* isolates with the IS element in their *adeRS* operon were genotyped by the pulsed-field gel electrophoresis (PFGE). Total DNA was prepared and PFGE was performed as described previously [39]. Endonuclease *Apa*I was used to digest the bacterial DNA, and then restriction fragments were analyzed by PFGE in 1% SeaKem Gold agarose gels (Cambrex Bio Science, Rockland, ME, USA) in 0.5× TBE buffer (45 mM Tris, 45 mM boric acid, 1.0 mM EDTA, pH 8.0) using a Bio-Rad CHEF-Mapper apparatus (Bio-Rad Laboratories, Hercules, CA). Gels were stained with ethidium bromide and photographed under ultraviolet light. Dendrograms showing percentage similarity were prepared with the Molecular Analyst Fingerprinting, Fingerprinting Plus and Fingerprinting DST Software (Bio-Rad Laboratories) and were compared using the UPGMA clustering method as proposed in previous studies [10], [39]. Isolates with similarity over 90% were considered to be identical; isolates with below 70% similarity (>3 band difference) were considered to be non-clonally related, using previously described criteria [39].

### Measurement of β-galactosidase activity

β-galactosidase activity assay was modified from the method described by Miller (1992) [40]. Briefly, 0.5 mL bacterial suspension of 37°C overnight culture was placed on ice for 20 min, and then harvested by centrifugation at 8000×g for 5 min at 4°C. The pellets were re-suspended in 0.5 mL of Z buffer (60 mMNa_2_HPO_4_·7H_2_O, 40 mM NaH_2_PO_4_·H_2_O, 10 mM KCl, 1 mM MgSO_4_·7H_2_O, 50 mM β-mercaptoethanol, pH 7.0) and the OD_600_ of the resuspended cells was measured (blank against Z buffer). To lysis the cells, 0.1 mL of the cell suspension was mixed with 0.4 mL of Z buffer, 50 µL of chloroform, and 25 µL of 0.1% Sodium dodecyl sulfate, vortexed for 10 sec, and incubated at 28°C. At a time point defined as zero, 0.1 mL of ONPG (o-nitrophenyl-β-D-galactopyranoside) (Sigma-Aldrich) (4 g/L) was added to the lysed cell suspension. The reaction was stopped by adding 0.25 mL of 1 MNa_2_CO_3_ at a time defined as T and the OD was then measured at 420 nm and 550 nm using a Synergy HT multi-mode microplate reader (BioTek Instruments, Winooski, VT). The activity of β-galactosidase was calculated using the following equation: 1000×[OD_420_−(1.75×OD_550_)]/[(T (min)×0.2 (mL)×OD_600_)], expressed in Miller units.

### Nucleotide sequence accession number

The 3038 -bp DNA sequences of the *adeRS* operon of AB260 and AB293 corresponding to the IS*Aba1*-interrupted *adeS* gene were submitted to the GenBank database as accession number JQ690823 and Q690824, respectively. The 2941-bp DNA sequence of the *adeRS* operon of AB392 corresponding to the IS*Aba125*-interrupted *adeR* gene was submitted to the GenBank database as accession number JQ690825.

## Results

### Compatible antibiotics susceptibility with PFGE genotyping results in MRAB-C isolates

**Figure 1:**
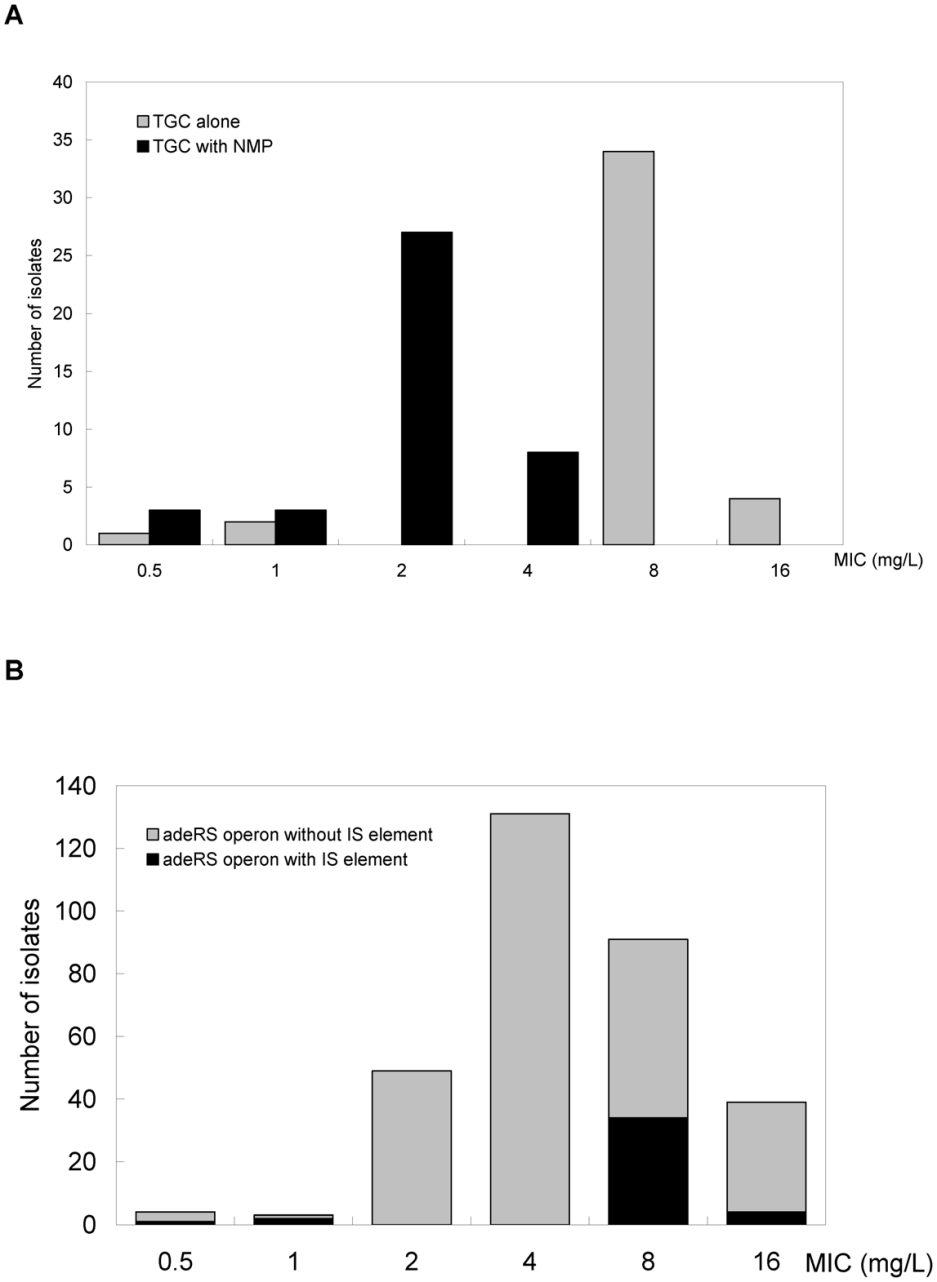
Tigecycline MIC profile of the clinical *A. baumannii* isolates. (A) Distribution of tigecycline MIC of the clinical *A. baumannii* isolates with (black bar) and without (grey bar) insertion of additional DNA in their *adeRS* operon (B) MIC to tigecycline alone (grey bar) and to tigecycline/NMP combination (black bar) for the 41 clinical *A. baumannii* isolates with insertion of additional DNA in their *adeRS* operon.

**Figure 2:**
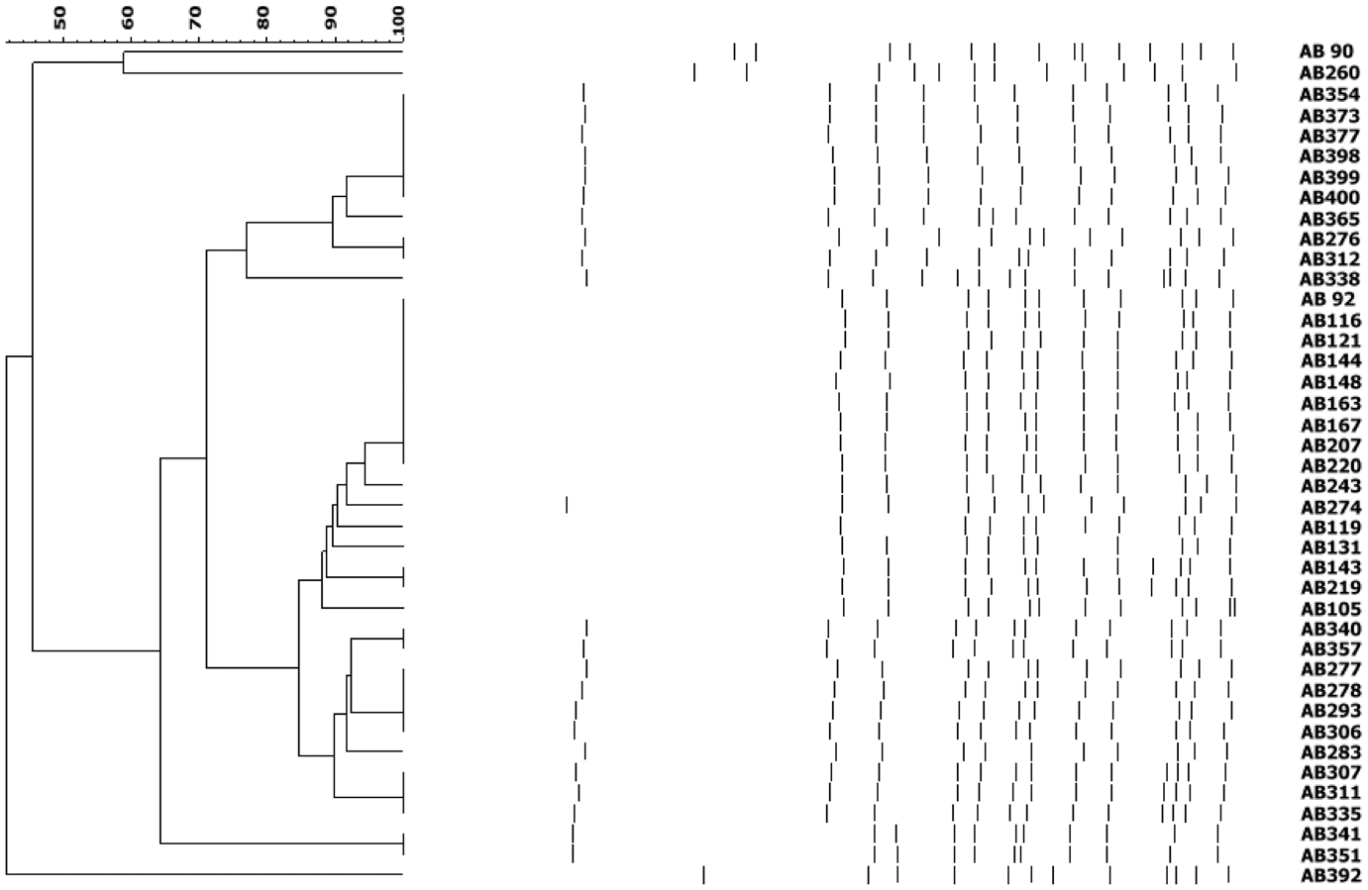
Pulsed-field gel electrophoresis (PFGE) for *Apa*I of 41 *A. baumannii* isolates with insertion of additional DNA in their *adeRS* operon. Dendrogram was generated by UPGMA clustering.

Antimicrobial susceptibility testing revealed that the 317 clinical MRAB-C isolates were resistant to all antibiotics tested in the Vitek-2 system. Using the FDA criteria for the agar dilution method, 130 (41%), 131 (41.3%), and 56 (17.7%) isolates were resistant, intermediate, and susceptible to TGC, respectively (Figure 1A). The full length of *adeRS* operon in each MRAB-C isolate was expected to be consistent in size (1.8 kb) after PCR amplification. However, the PCR products from 41 isolates were about 3.0 kb, which indicates additional DNA sequences within the *adeRS* operon. Among the 41 isolates, 38 (92.7%) isolates were resistant and 3 (7.3%) were susceptible to TGC. In a previous study, we demonstrated the expression of AdeABC efflux pump is significantly correlated with MIC of TGC in clinical isolates during the period [10]. To verify the effect of AdeABC efflux pump on TGC resistance, all MRAB-C isolates were tested for their MIC of TGC in the presence of NMP, an efflux pump inhibitor (Figure 1B). On plates with NMP, their E-test MIC (0.5–4 mg/L) of TGC showed 2–8 folds lower values than those without NMP (8–16 mg/L). TGC resistance was therefore related to AdeABC efflux pump overexpression in these isolates. After comparing the genotype patterns by pulsed field gel electrophoresis (PFGE) with *Apa*I-digestion, the 41 isolates were classified into 3 clusters and 3 sporadic clones based on PFGE criteria (Figure 2). The genotypic grouping result was compatible with phenotypes of TGC susceptibility. Two major clusters (AB354-AB338 and AB92-AB335 in Figure 2) and one minor cluster (AB341 and AB351) contained 38 (92.7%, 38/41) of all the MRAB-C isolates that were all TGC resistant. The remaining 3 sporadic clones (AB90, AB260 and AB392) were TGC susceptible.

### IS elements within the *adeRS* operon found by sequencing analysis

**Figure 3:**
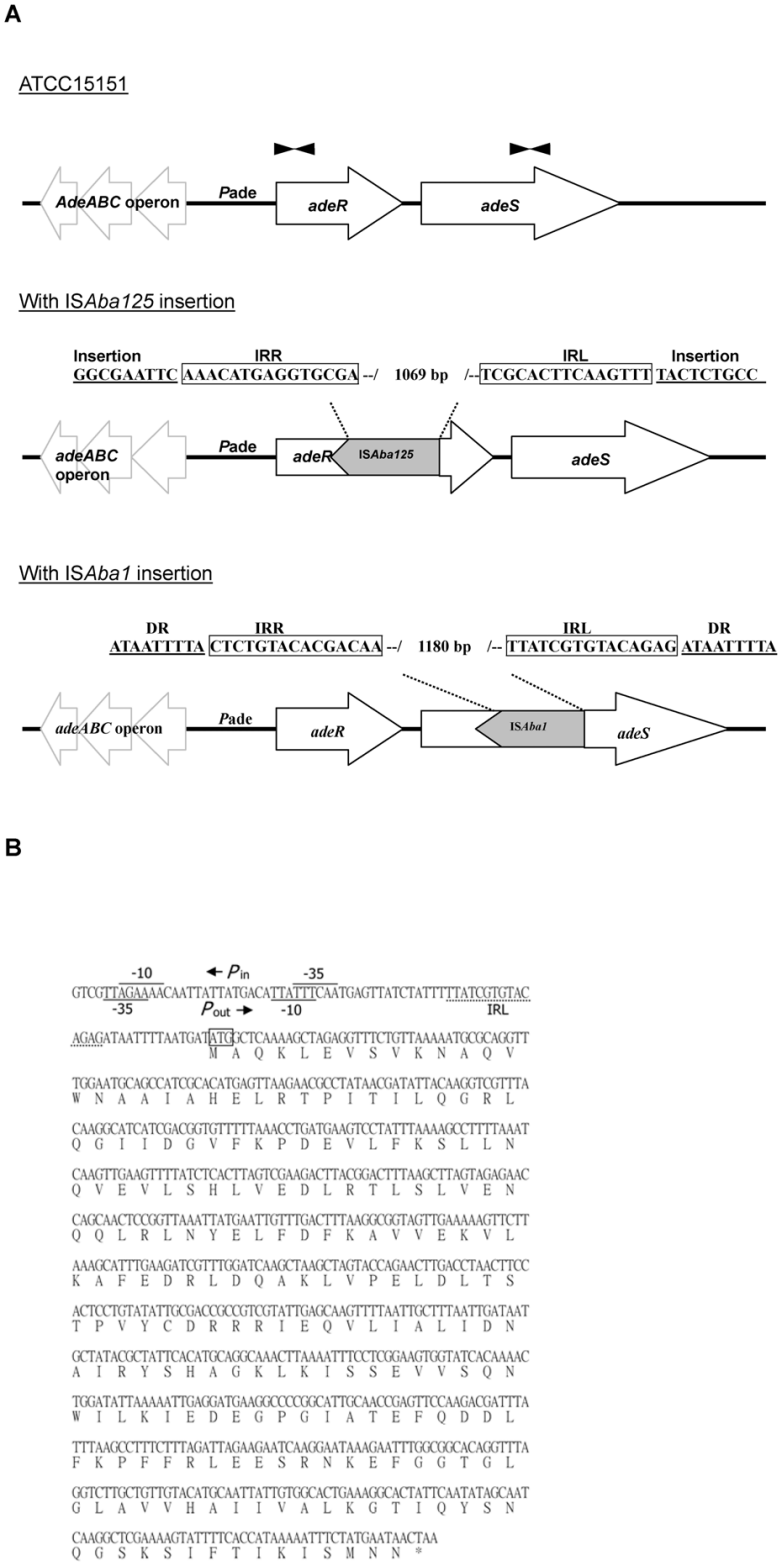
Alteration of the *adeRS* operons by insertion of IS*Aba125* or IS*Aba1* in multidrug resistant *A. baumannii* isolates. (A) Genetic scheme of wide type *adeRS* operon in ATCC 15151 and two insertion mutations. IS*Aba*125 disrupts the *adeR* gene and IS*Aba1* disrupts the adeS gene in clinical multidrug resistant *A. baumannii* isolates. Open arrows indicate coding sequences and direction of transcription. *P*ade is the promoter of the *adeABC* operon. Closed arrowheads indicate the position and orientation of the primers used in quantitative *adeR* and *adeS* real-time PCR (all positions are according to accession no. JQ690823-5 in GenBank). IRR, inverted repeat-right; IRL, inverted repeats-left; DR, direct repeat. (B) Presumed promoters in IS*Aba1* and downstream coding sequence of the truncated AdeS. The deduced amino-acid sequence is designated in a single letter code below the nucleotide sequence and the star sign indicates stop codon. The inverted repeat sequences (IRL) of IS*Aba1* are dash-underlined. The −35 and −10 motifs of promoters are indicated as numbers. *P*in is the promoter for transposase gene in IS*Aba1*, and *P*out is the promoter for the truncated AdeS derived from IS*Aba1* insertion. The truncated AdeS start codon is boxed.

To determine the additional DNA insertion in *adeRS* operon, direct sequencing of *adeRS* operon PCR products showed the presence of the IS*Aba125* element within the *adeR* gene in AB392 isolates and the IS*Aba*1 element within the *adeS* gene in the remaining 40 strains (Figure 3A). The IS*Aba125* element was 1,087-bp in length. It contained a 969-bp open reading frame (ORF) encoding a putative transposase, and was bounded by 15-bp inverted repeat sequences which are common to members of the IS*30* group. Two 9-bp individual sequences (GGCGAATTC and TACTCTGCC) flanked the IS*Aba125* at the predicted insertion site in *adeR* gene of AB392. The presence of IS*Aba125* mediated disruptive events on the *adeR* gene in AB392, resulting in the loss function of *adeR*, and maybe affecting the expression of the AdeABC efflux pump system. The IS*Aba1* element was 1180-bp in length, containing two consecutive and overlapping ORFs, 567-bp ORF1 and 535-bp ORF2. It was therefore likely that a frame-shift is necessary to generate a unique protein as functional transposase. The IS*Aba1* was bounded by 15-bp inverted repeat sequences which are common to members of the IS*4* family (Figure 3A). A 9-bp duplication (ATAATTTTA) flanked IS*Aba1* at the predicted insertion site in *adeS* gene of the other isolates. The presence of IS*Aba1* mediated disruptive events on the *adeS* gene, resulting in the loss function of *adeS* gene, and maybe affecting the expression of the AdeABC efflux pump system. However, we noted the most striking difference in the distribution of TGC MIC in the isolates. Only 2 strains (AB090 and AB260) were TGC susceptible and the remaining 38 isolates were TGC resistant. Hence, it was necessary to investigate whether the phenomenon was caused by DNA mutation in the promoter region or in *adeRS* operon. Nucleotide sequences of the promoter region and the *adeRS* operon with IS*Aba1* insertion were analyzed by multiple sequence alignment. DNA sequences of the promoter region and the destroyed *adeS* gene were identical among the 38 TGC resistant isolates. However, two point mutations (Met197Ile) and (Ser200Cys) in *adeR* gene were identified in all isolates, except for the two TGC sensitive isolates.

### Transcript analysis of *adeABC* operon and *adeRS* operon

As the synergistic effect of NMP on TGC susceptibility described above, the TGC resistance may be associated with up-regulation of the AdeABC efflux pump in those isolates with their *adeS* gene destroyed by IS*Aba1*. We investigated the transcription activity of *adeRS* operon and *adeABC* operon in representative isolates with various TGC MICs using quantitative real-time RT-PCR (Table 3). The two isolates (AB039 and AB096) were discussed in our previous research [10], [41], [42]. They both have higher MIC of TGC than strain ATCC 15151. The MICs of TGC were 2 mg/L and 16 mg/L for AB39 and AB96, respectively. Although the detail mechanism was not explored, we have demonstrated the higher MIC is proportionally correlated with the *adeB* expression level. We used these two strains simply to show the consistency of our experiment. We were still not able to identify the exact cause for their difference. In this manuscript, nonetheless, we focused on the group of isolates with an insertion element.

**Table 3:** Relationship between tigecycline MICs and expression of *adeRS* and *adeABC* operons in clinical strains of *A. baumannii*.

			Differential quantification of gene expression (fold)			
Strains	Strains	TGC MIC (mg/L)	*adeR*	*adeS*	*adeA*	*adeB*
Without insertion	ATCC 15151	≦0.5	1.00	1.00	1.00	1.00
	AB39	2	1.50±0.73	0.65±0.2	24.15±5.95	42.64±22.49
	AB96	16	1.55±0.15	0.67±0.05	285.75±106.12	622.03±189.32
With insertion	AB392 (IS*Aba125*)	≦0.5	1.21±0.25	0.08±0.02	0.55±0.33	1.05±0.64
	AB260 (IS*Aba1*)	1	1.87±0.14	2.82±0.88	1.02±0.54	1.71±0.59
	AB293 (IS*Aba1*)	16	2.45±0.98	2.12±0.35	51.32±9.21	81.75±68.78

To quantify the *adeRS* operon expression in strains with various IS element insertion, the first set of PCR primers for *adeR* gene was targeting the region between its promoter and the IS*Aba125* insertion site in AB392 (Figure 3A). The second set of PCR primers was targeting the terminal region of *adeS* gene after the IS*Aba1* insertion site in AB293 (Figure 3A). We first compared the *adeB* gene expression in TGC resistant isolates (AB096 and AB293) with the proposed basal expression in control strain ATCC 15151. The expression of *adeB* gene increased 622 times and 82 times in AB096 and AB293, respectively. In contrast, only a 42-fold, 1.1-fold and 1.7-fold increase in the *adeB* gene expression was observed in TGC susceptible clinical strains, AB039, AB392 and AB260, respectively. The *adeA* gene showed the same increasing expression pattern in TGC resistant clinical isolates as the *adeB* gene. All the TGC susceptible isolates (ATCC15151, AB260 and AB392) had less transcription of *adeA* and *adeB* consistently. In contrast, the transcription levels of the *adeA* and *adeB* genes in TGC resistant isolates were significantly higher than those in TGC susceptibility isolates (Mann-Whitney Test, *P* value <0.05). We tried to correlate the overexpression of *adeA* and *adeB* genes with their regulatory compartment by quantifying the transcripts of *adeR* and *adeS* genes in the TGC resistant isolates. The level of transcription of *adeR* in AB260 (1.87±0.14) and AB293 (2.45±0.98) was not significantly different (Mann-Whitney Test; *P* value = 0.38). However, the *adeS* gene transcription in AB392 was 12.5-fold lower than in the ATCC 15151 control strain. Lower *adeS* transcription might result from the polar effect after an IS*Aba125* insertion in AB392. The IS*Aba1* insertion also resulted in the same polar effect after an IS*Aba1* insertion in AB260, but did not cause the same polar effect to reduce the *adeS* gene transcription in AB293. Our findings suggested that an extra promoter within the IS*aba1* element of AB293 constitutively drives the transcription for the remaining *adeS* gene fragment.

### A truncated AdeS predicted by DNA sequencing

To determine the structure and function domain of AdeS, we analyzed the full adeS gene sequence in ATCC 15151 by the NCBI tool and the transmembrane domain prediction tool. The N-terminus of the AdeS contained two transmembrane segments (amino acid positions 9–36 and 58–85), which suggests that AdeS is a membrane anchored protein. The C-terminus of AdeS after the second transmembrane segment was the transmitter compartment containing a phosphorylatable HisKA domain and an ATP -binding domain. The insertion site of IS*Aba*1 was located between the second transmembrane segment and the transmitter compartment in AB293. Also, a *P*out promoter overlapping with *Pin* promoter was found near left inverted repeat sequence (IRL) within the IS*Aba1* element (Figure 3B). By analyzing the downstream sequences of the *P*out promoter, we predicted an ORF encoding a protein with 229 amino acids by ORF finder. The amino acid sequence of the predicted protein was the same as the carboxyl-terminal transmitter domain of AdeS protein. We deduced a truncated AdeS driven by the *P*out promoter in the IS*Aba1* element (Figure 3B). The predicted protein contained neither transmembrane helices nor a signal peptide. It was suggested to be a truncated protein within the cytoplasm. The truncated AdeS shared 100% amino acid identity with the kinase sensor protein annotated as a hypothetical protein of the MRAB-C strain 1656-2 (GenBank accession number ADX03920). We further analyzed the biological function of the truncated AdeS protein by investigating its possible relationship with two point mutations of AdeR in determining TGC resistance.

### Acquired TGC resistance induced by coexistence of the truncated AdeS and mutated AdeR

**Figure 4:**
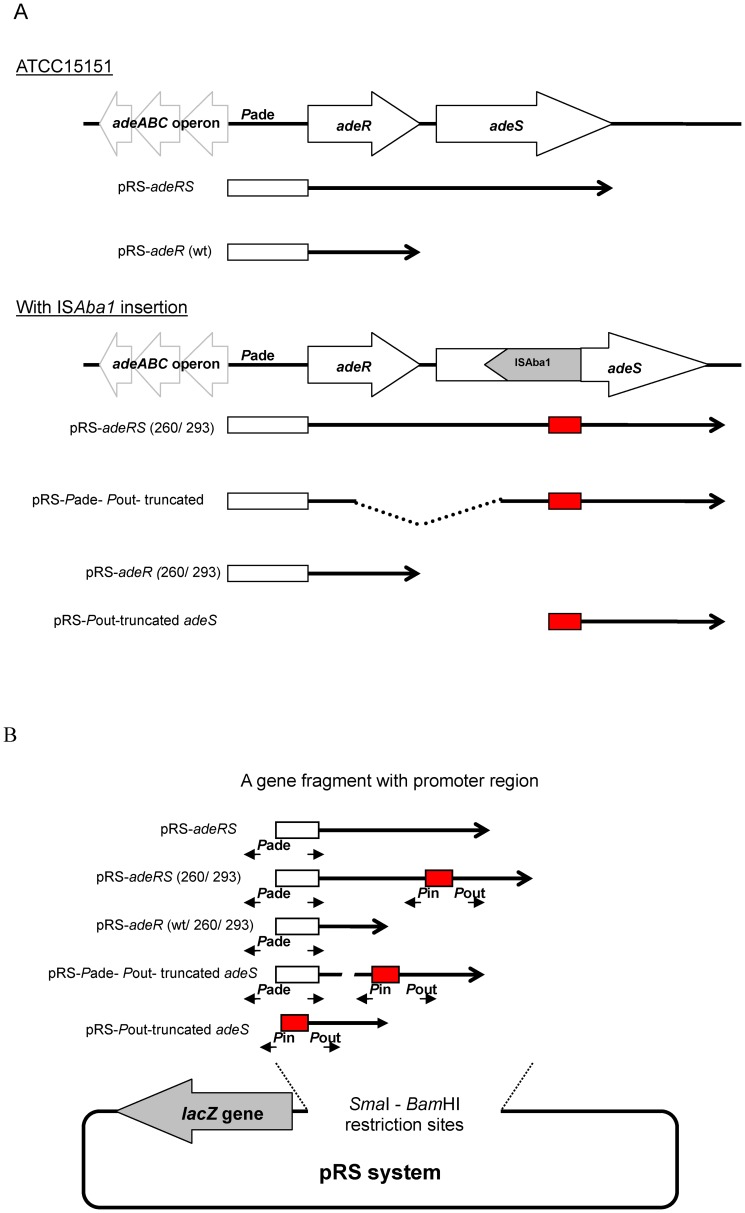
Construction of the pRS recombinant series. (A) Strategies for obtaining a series of DNA fragments containing promoter region of *adeABC* operon and a series of *adeRS* operon variants from wild type (wt) ATCC 15151 and drug resistant strains (260, 293) by PCR. Open arrows denote coding sequences and indicate the direction of transcription in the bacterial genome. The white rectangle box denotes the region of *P*ade promoter (*P*ade and *P*re). The red rectangle box denotes the promoter region in IS*Aba1* (*P*in and *P*out). Arrows following the rectangle boxes together indicate the PCR fragments used to generate pRS recombinant series. (B) Construction map of the promoter-less *lacZ* gene of pRS system. The pRS system was derived from pS01, which remove *lacI^q^*-P_T7_ promoter by engineered *Sma*I and *Bam*HI cutting. The pRS system was obtained and used for cloning PCR fragments into upstream of the promoter-less *lacZ* gene of pRS system between *Sma*I and *Bam*HI restriction sites. The rectangle box indicates the region of promoters. The region of *P*ade promoter region contains two overlapped promoters in opposite direction (*P*ade and *P*re). Two overlapped promoters, *P*in and *P*out, were in the region of promoter region in IS*Aba1*. The expression level of *lacZ* gene represented for the activity of promoter of *adeABC* operon or *P*in promoter of IS*Aba1*.

To further assess the relationship between the truncated AdeS and mutated AdeR in TGC resistant isolates, DNA fragments of the full length *adeRS* operon and the *adeR* gene from different strains (wt, AB260 and AB293) were cloned into the pRS system to construct pRS-*adeRS* (wt, AB260, AB293) and pRS-*adeR* (wt, AB260, AB293), respectively (Figure 4A). The predicted truncated *adeS* operon of AB293 was also cloned into pRS to generate pRS-Pout-truncated *adeS*. The pRS-*adeRS* (AB293) was digested with *Sph*I and was self-ligated to generate pRS-*P*ade-*P*out-truncated *adeS*. The *adeRS* opreon of ATCC 15151 was destroyed to generate a mutant bacterial strain (ABmut02) by homologous recombination with a suicide plasmid. These recombinants and the ABmut02 mutant strain were used to assess the regulation of AdeABC efflux pump expression. The recombinant plasmids were transformed respectively into ATCC 15151 and ABmut02 to obtain the transformed strains. The transformed strains were then tested for their antimicrobial susceptibilities. Results of the antibiotic susceptibility assays were summarized in Table 4. Assays on ATCC15151 wild type strain showed an 8-fold TGC MIC increase after transforming ATCC 15151 with pRS-*adeRS* (AB260), pRS-*adeRS* (AB293), pRS-Pade-Pout-truncated *adeS* or pRS-Pout-truncated adeS. The AdeRS-destroyed mutant ABmut02 shared the same TGC MIC with wild type ATCC 15151 after transforming with the pRS-*adeRS* (AB260) or pRS-*adeRS* (AB293). However, contrary to expectation, transformation with pRS-*P*ade-*P*out-truncated_*adeS* or pRS-*P*out-truncated_*adeS* did not show higher TGC MIC in ABmut02. We demonstrated that the truncated AdeS must cooperate with AdeR to induce TGC resistance. And the two point mutations in AdeR were proven to have no significant effect on its interaction with the truncated AdeS. To determine whether the multidrug resistance was directly induced by the truncated AdeS in the pRS system, we disrupted the start codon of the truncated AdeS by site directed mutagenesis. We found 16-fold lower TGC MIC and multidrug MIC reduction in ATCC 15151 transformed with the start codon disrupted mutant, pRS-Pout-truncated_adeS (SDM). Accordingly, we suggest that truncated AdeS is associated with acquired TGC and multidrug resistance.

**Table 4:** Antimicrobial susceptibilities of the various *A. baumannii* transformants.

		MIC (mg/liter) of				
Strains	with plasmid	TGC	TGC+ NMP	FEP	CIP	LEV
ATCC 15151 (wt *adeRS*)	no plasmid	0.25	0.25	2	≦0.25	≦0.12
	pS01	0.125	0.0625	2	≦0.25	≦0.12
	pRS-*adeRS* (wt)	0.25	0.125	2	≦0.25	≦0.12
	pRS-*adeRS* (AB260)	2	0.25	16	1	0.25
	pRS-*adeRS* (AB293)	2	0.25	16	1	0.25
	pRS-*adeR* (wt)	0.25	0.125	2	≦0.25	≦0.12
	pRS-*adeR* (AB260)	0.25	0.125	2	≦0.25	≦0.12
	pRS-*adeR* (AB293)	0.25	0.125	2	≦0.25	≦0.12
	pRS-P*ade*-P*out*-truncated *adeS*	2	0.25	16	0.5	0.25
	pRS-P*out*-truncated *adeS*	2	0.25	16	0.5	0.25
	pRS-P*out*-truncated *adeS* (SDM)	0.25	0.125	2	≦0.25	≦0.12
ABmut02 (*adeRS* destruct)	no plasmid	0.25	0.25	2	≦0.25	≦0.12
	pS01	0.25	0.25	2	≦0.25	≦0.12
	pRS-*adeRS* (wt)	0.125	0.0625	2	≦0.25	≦0.12
	pRS-*adeRS* (AB260)	2	0.25	16	0.5	0.25
	pRS-*adeRS* (AB293)	2	0.25	32	1	0.25
	pRS-*adeR* (wt)	0.125	0.0625	2	≦0.25	≦0.12
	pRS-*adeR* (AB260)	0.125	0.0625	2	≦0.25	≦0.12
	pRS-*adeR* (AB293)	0.125	0.0625	2	≦0.25	≦0.12
	pRS-P*ade*-Pout-truncated adeS	0.125	0.0625	2	≦0.25	≦0.12
	pRS-P*out*-truncated *adeS*	0.125	0.0625	2	≦0.25	≦0.12
	pRS-P*out*-truncated *adeS* (SDM)	0.125	0.0625	2	≦0.25	≦0.12

### Quantifying the *P*ade promoter activity by the β-galactosidase assay

**Figure 5:**
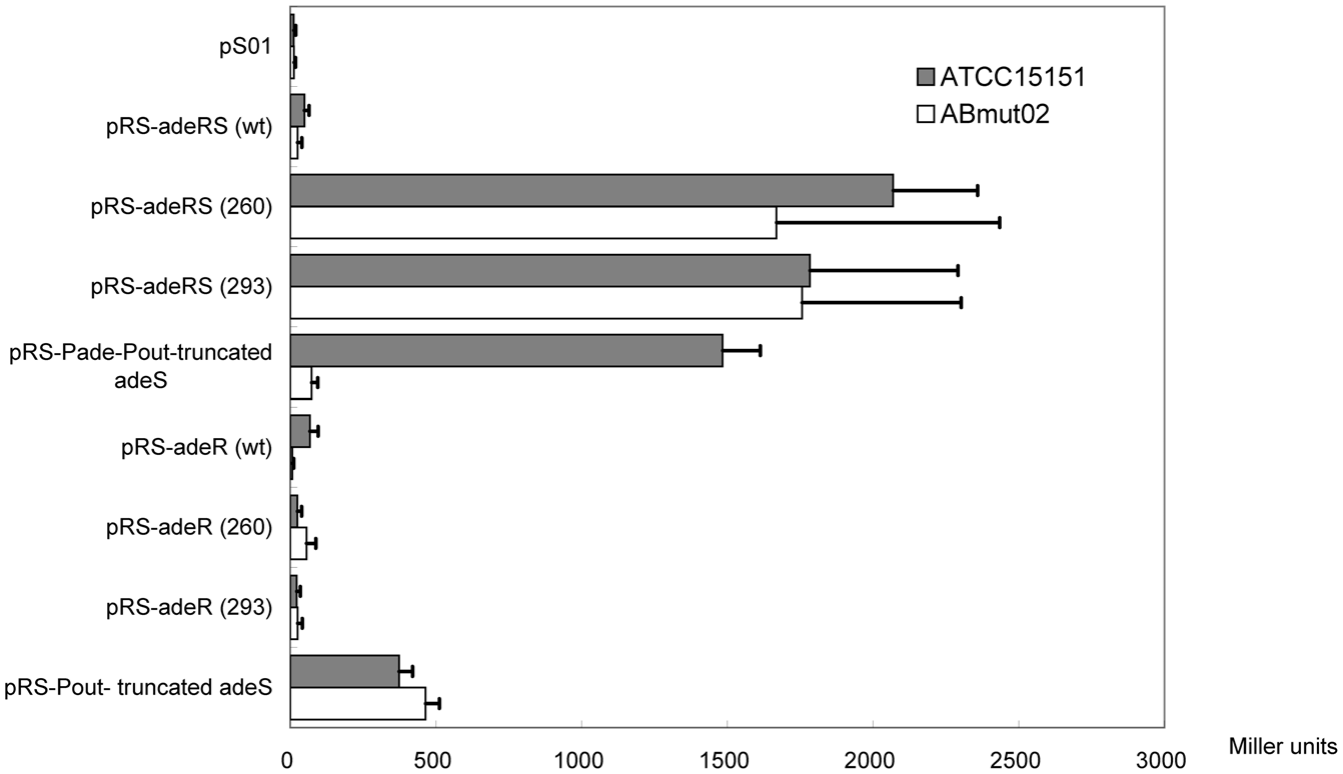
β-galactosidase activities of transformed strains with various pRS recombinants. ATCC 15151 and ABmut02 cells transformed with various pRS recombinants were grown in LB medium at 37°C overnight. The measurement of β-galactosidase activity was repeated at least three times, and the mean values of one representative experiment performed in triplicate are reported (error bars indicate the standard deviation). The measurement of β-galactosidase activity represents for the activity driven by either *P*ade of *adeABC* operon or *P*in of IS*Aba1*.

To further differentiate the promoter effect from either *P*ade of *adeABC* operon or *P*in of ISaba1, we quantified *adeA* and *adeB* gene expression in all the transformants listed in Table 4. We constructed a *lacZ* gene reporter system instead of laborious quantification RT-PCR of *adeA* and *adeB*. The *lacZ* gene was cloned as a substitute for the *adeABC* gene in the pRS series (Figure 4B). Then we were able to assay the promoter effect by directly quantifying β-galactosidase activity. The β-galactosidase activities in ATCC 15151 strain with pS01 (13±7 units) and in ABmut02 strain with pS01 (12±6 units) were measured as background. In ATCC 15151, β-galactosidase activities increased about 172-fold (2068±290 units), 149-fold (1783±508 units) and 123-fold after transformation with pRS-*adeRS* (260), pRS-*adeRS* (293), and pRS-Pade-Pout-truncated *adeS*, respectively (Mann-Whitney Test; *P* value <0.05). In ABmut02, β-galactosidase activities also increased about 128-fold (1669±766 units) and 135-fold (1757±546 units) after transformation with pRS-*adeRS* (260) and pRS-*adeRS* (293), respectively (Mann-Whitney Test; *P* value <0.05) (Figure 5). Transformation of pRS-*P*ade-*P*out-truncated *adeS* can only increase β-galactosidase activitiy by 20 fold while AdeR is lacking in ABmut02 strain (Mann-Whitney Test; *P* value <0.05). The β-galactosidase activity did not increase while being transformed with the recombinant plasmids expressing adeR only (wt, 260, 293) into either ATCC 15151 or ABmut02 strains. These results suggested that the expression of the *adeABC* efflux pump was directly controlled by the two-component system, truncated AdeS and AdeR. The expression of β-galactosidase activity was significantly higher in ATCC15151 with pRS-*Pade*-*P*out-truncated *adeS* than with pRS-*P*out-truncated *adeS* (Mann-Whitney Test; *P* value <0.05). And the lower expression of β-galactosidase activity was observed in ABmut02 with pRS-*P*ade-*P*out-truncated *adeS* than with pRS-*P*out-truncated *adeS* (Mann-Whitney Test; *P* value <0.05). These results suggested that AdeR protein is necessary for stimulating *Pade* promoter and enhancing the expression of the *adeABC* efflux pump. In order to rule out the possibility of *P*in promoter of IS*Aba1* on enhancing the expression of *adeABC* operon, we only increased the β-galactosidase activity of ATCC 15151 and ABmut02 transformed with the *P*in containing plasmid pRS-*P*out-truncated_*adeS* by 31-fold (374±46 units) and 36-fold (465±48 units), respectively. The β-galactosidase assay successfully quantified the significantly higher *lacZ* activity in the strain transformed with pRS-*adeRS* compared with pRS-*P*out-truncated *adeS*. The biological function of truncated adeS was further demonstrated besides converting the MIC presentation, and the truncated adeS with coexistence of adeR can constitutively stimulate the promoter *P*ade to express the *lacZ* gene.

## Discussion

Antibiotic resistance is a major challenge in treating infections caused by *A. baumannii* [1]. The *adeABC* operon is located in chromosomal DNA and it endorses the intrinsic low-level expression in *A. baumannii*. [3], [7]. The expression of AdeABC efflux pump is tightly regulated by the two-component regulatory system AdeR-AdeS, encoded by the *adeRS* operon that is located upstream of *adeABC* operon and transcribed in the opposite direction [3], [7], [14].. The overexpression of the AdeABC efflux pump, responsible for reducing accumulation of many antibiotics, is an efficient mechanism for multidrug resistance in *A. baumannii* [43]. AdeS is a membrane-integrated sensor protein with a histidine kinase domain which converts and transfers environmental stimuli to a response regulator AdeR for regulating the AdeABC gene expression [3], [7]. On the basis of our current data, we proposed an N-terminal truncated AdeS generated by the Pout promoter within the IS*Aba1* insertion of *adeS* gene. The truncated AdeS protein was able to activate AdeR and then enhance the *adeABC* gene expression.

Ruzin et al. [9] reported they found the disruption of AdeS by the insertion sequence IS*Aba1* in two TGC-resistant isolates of *Acinetobacter calcoaceticus*–*A. baumannii* complex. The AdeABC pumps were overexpressed in these strains and associated with TGC resistant. However, the prevalence, genotype and mechanism of the strain with an IS*Aba1* insertion in *adeS* gene had not been well investigated. In this study, we found 41 of the 317 isolates contained DNA insertions in their *adeRS* operon. Besides the IS*Aba1* insertion of adeS gene in 40 isolates as presumed, the remaining one isolate was found with IS*Aba125* insertion in its *adeR* gene. Compared with the TGC susceptibility phenotype, we found that 38 of the above 40 isolates accounted for 29.2% (38/130) of the TGC resistant isolates, and they demonstrated differences in the PFGE patterns. This result may indicate a common ancestor shared by these isolates with IS*Aba1* insertion in *adeS* gene.

Previous studies including ours have indicated that the sequence of AdeR and AdeS are widely variable in clinical isolates, but several mutations are still recommended for causing overexpression of the AdeABC efflux pump [10], [41], [42]. Most of the publications describe point mutations in the sensor domain (Asp30Gly), in the linker domain (Aln94Val) between the recognition domain and the histidine box, and in the histidine box (Tro153Met) of the AdeS [14], [44]. A mutation (Pro116Leu) in AdeR is also reported to cause *adeABC* operon overexpression likely due to altering the structural conformation of the regulator [14]. In addition, AdeR mutation (Aln91Val) and AdeS mutation (Gly103Asp) occur concurrently in a susceptibility conversion strain after exposure to TGC. However, the detail interaction among these mutations has not been ascertained [42]. Although we found two new mutations (Met197Ile) and (Ser200Cys) on the DNA binding domain of AdeR protein of all TGC resistant strains included in this study, strain AB260 with the same mutations showed lower expression level of the *adeABC* operon and was susceptible to TGC. However, the transformed strains with pRS-adeRS(AB293) and with pRS-adeRS(AB260) were presented with the presumed higher MIC to TGC. We therefore suggested that the *adeABC* operon expression in the susceptible strain AB260 might be inhibited by some unknown mechanism. Future studies will focus on analyzing the inhibition mechanism of *adeABC* operon expression in the TGC susceptibility strain.

IS*Aba1* copies were found in the chromosome and plasmids, which have been known to be related to antibiotic resistance only in *Acinetobacter* spp [27]. Previous genome-wide studies on evaluating the drug resistance mechanism in clinical *A. baumannii* isolates found multidrug resistant isolates carry more IS*Aba1* elements copies (9 to 428) than clinical susceptible strains and laboratory strains [21], [45], [46], [47]. The IS*Aba1* element flanks an antibiotic resistant gene and is involved in the overexpression of the genes from an outward-facing promoter, *P*out in the IS element. Another example, disruption of an antibiotics influx pump protein, designated CarO, has also been reported by the IS*Aba1* element insertional inactivation [34]. Other IS*Aba1* insertions are polymorphic and are thus unlikely to be directly related to drug resistance in these strains [48]. Some IS*Aba1* insertion locations are also found in or adjacent to hypothetical protein genes. Previous genome-wide studies have identified an IS*Aba1* insertion in the *adeS* gene and even a presumed sensor kinase protein [21], [45]. In this study, a similar IS*Aba1* insertion into *adeS* was demonstrated to generate a truncated AdeS which shared the same amino acid sequence as the predicted sensor kinase protein of the genome-wide study [45]. We therefore suggested that the *A. baumannii* strain with a truncated *adeS* gene through IS*Aba1* insertion may have a worldwide distribution and require further investigation.

Instead of laboriously comparing the possible genomic difference among *A. baumannii* strains, we converted the reference strain ATCC 15151 to an AdeRS-knockout laboratory strain, ABmut02. A series of pRS recombinants carrying different *adeRS* fragments and the *lacZ* reporter gene were then able to be analyzed for their regulatory effect in the mutant strain. By directly quantifying the expression of the *lacZ* gene, we can compare the triggering effect of various *adeRS* operons on the *adeABC* promoter. Because *adeR* and *adeS* operon array sequentially after the same promoter, we did not obtain a laboratory strain solely with *adeR* gene disruption. The MIC of TGC can be as high as 16 mg/L for clinical resistant isolates. However, MICs of TGC were only mildly elevated (MIC = 2 mg/L, 8-folds) after transforming the truncated AdeS into ATCC 15151 and its adeRS-knockout derivative. The partially resistance-converting result suggests there are other mechanisms affecting the AdeABC efflux system. We presume some inhibitors offset the truncated AdeS effect in ATCC 15151 and AB260, but not in AB293. This inhibitor proposal may explain why isolate AB260 only demonstrates moderate MIC to TGC (1 mg/L) instead of MIC 16 mg/L in AB293. In addition, boosting AdeS by transforming a plasmid encoded the truncated *adeS* operon into ATCC 15151 did convert its susceptibility to TGC, while the same plasmid alone was unable to overcome the proposed inhibitor in ATCC 15151 mutant (ABmut02) with the destructed *adeRS*. The observed alteration of MIC to TGC for those artificial transformants supports our proposal indirectly. However, we need more direct evidence to prove this proposal. Moreover, an extraordinary efflux pump or other resistance mechanism may coexist to result in the aberrantly high MIC to TGC in AB293.

In this report, we have shown the distribution of multidrug resistant *A. baumannii* with the IS*Aba1* insertion in *adeS* gene in Taiwan. We have also demonstrated the existence of a truncated AdeS and its biological function. The truncated AdeS is able to induce multidrug resistance by constitutively stimulating the expression of AdeABC efflux pump through motivating the AdeR to interact with and activate the *adeABC* promoter. Our findings not only confirm the previously presumed additional *adeS* gene after IS*Aba1* insertion, but also further characterize it as another mechanism for IS*Aba1*-related antibiotic resistance. As the two-component system is a vital structure of the bacteria and is used to adapt to changing environmental conditions, insight and applications resulting from studies on *A. baumannii* will probably be broadly applicable to other bacteria.

## References

[1] Peleg AY, Seifert H, Paterson DL (2008) Acinetobacter baumannii: emergence of a successful pathogen. Clin Microbiol Rev 21: 538–582. 18625687 10.1128/CMR.00058-07 PMC2493088

[2] Dijkshoorn L, Nemec A, Seifert H (2007) An increasing threat in hospitals: multidrug-resistant Acinetobacter baumannii. Nat Rev Microbiol 5: 939–951. 18007677 10.1038/nrmicro1789

[3] Coyne S, Courvalin P, Perichon B (2011) Efflux-mediated antibiotic resistance in Acinetobacter spp. Antimicrob Agents Chemother 55: 947–953. 21173183 10.1128/AAC.01388-10 PMC3067115

[4] Gordon NC, Wareham DW (2009) A review of clinical and microbiological outcomes following treatment of infections involving multidrug-resistant Acinetobacter baumannii with tigecycline. J Antimicrob Chemother 63: 775–780. 19158109 10.1093/jac/dkn555

[5] Cortez-Cordova J, Kumar A (2011) Activity of the efflux pump inhibitor phenylalanine-arginine beta-naphthylamide against the AdeFGH pump of Acinetobacter baumannii. Int J Antimicrob Agents 37: 420–424. 21377839 10.1016/j.ijantimicag.2011.01.006

[6] Yahav D, Lador A, Paul M, Leibovici L (2011) Efficacy and safety of tigecycline: a systematic review and meta-analysis. J Antimicrob Chemother 66: 1963–1971. 21685488 10.1093/jac/dkr242

[7] Wieczorek P, Sacha P, Hauschild T, Zorawski M, Krawczyk M, et al (2008) Multidrug resistant Acinetobacter baumannii–the role of AdeABC (RND family) efflux pump in resistance to antibiotics. Folia Histochem Cytobiol 46: 257–267. 19056528 10.2478/v10042-008-0056-x

[8] Vila J, Marti S, Sanchez-Cespedes J (2007) Porins, efflux pumps and multidrug resistance in Acinetobacter baumannii. J Antimicrob Chemother 59: 1210–1215. 17324960 10.1093/jac/dkl509

[9] Ruzin A, Keeney D, Bradford PA (2007) AdeABC multidrug efflux pump is associated with decreased susceptibility to tigecycline in Acinetobacter calcoaceticus-Acinetobacter baumannii complex. J Antimicrob Chemother 59: 1001–1004. 17363424 10.1093/jac/dkm058

[10] Sun JR, Chan MC, Chang TY, Wang WY, Chiueh TS (2010) Overexpression of the adeB gene in clinical isolates of tigecycline-nonsusceptible Acinetobacter baumannii without insertion mutations in adeRS. Antimicrob Agents Chemother 54: 4934–4938. 20696871 10.1128/AAC.00414-10 PMC2976115

[11] Ruzin A, Immermann FW, Bradford PA (2010) RT-PCR and statistical analyses of adeABC expression in clinical isolates of Acinetobacter calcoaceticus-Acinetobacter baumannii complex. Microb Drug Resist 16: 87–89. 20438348 10.1089/mdr.2009.0131

[12] Damier-Piolle L, Magnet S, Bremont S, Lambert T, Courvalin P (2008) AdeIJK, a resistance-nodulation-cell division pump effluxing multiple antibiotics in Acinetobacter baumannii. Antimicrob Agents Chemother 52: 557–562. 18086852 10.1128/AAC.00732-07 PMC2224764

[13] Coyne S, Rosenfeld N, Lambert T, Courvalin P, Perichon B (2010) Overexpression of resistance-nodulation-cell division pump AdeFGH confers multidrug resistance in Acinetobacter baumannii. Antimicrob Agents Chemother 54: 4389–4393. 20696879 10.1128/AAC.00155-10 PMC2944555

[14] Marchand I, Damier-Piolle L, Courvalin P, Lambert T (2004) Expression of the RND-type efflux pump AdeABC in Acinetobacter baumannii is regulated by the AdeRS two-component system. Antimicrob Agents Chemother 48: 3298–3304. 15328088 10.1128/AAC.48.9.3298-3304.2004 PMC514774

[15] Szurmant H, White RA, Hoch JA (2007) Sensor complexes regulating two-component signal transduction. Curr Opin Struct Biol 17: 706–715. 17913492 10.1016/j.sbi.2007.08.019 PMC2175030

[16] Cheung J, Hendrickson WA (2010) Sensor domains of two-component regulatory systems. Curr Opin Microbiol 13: 116–123. 20223701 10.1016/j.mib.2010.01.016 PMC3078554

[17] Essen LO, Mailliet J, Hughes J (2008) The structure of a complete phytochrome sensory module in the Pr ground state. Proc Natl Acad Sci U S A 105: 14709–14714. 18799745 10.1073/pnas.0806477105 PMC2567182

[18] Yang X, Kuk J, Moffat K (2008) Crystal structure of Pseudomonas aeruginosa bacteriophytochrome: photoconversion and signal transduction. Proc Natl Acad Sci U S A 105: 14715–14720. 18799746 10.1073/pnas.0806718105 PMC2567202

[19] Mern DS, Ha SW, Khodaverdi V, Gliese N, Gorisch H (2010) A complex regulatory network controls aerobic ethanol oxidation in Pseudomonas aeruginosa: indication of four levels of sensor kinases and response regulators. Microbiology 156: 1505–1516. 20093290 10.1099/mic.0.032847-0

[20] Gorisch H (2003) The ethanol oxidation system and its regulation in Pseudomonas aeruginosa. Biochim Biophys Acta 1647: 98–102. 12686116 10.1016/s1570-9639(03)00066-9

[21] Adams MD, Chan ER, Molyneaux ND, Bonomo RA (2010) Genomewide analysis of divergence of antibiotic resistance determinants in closely related isolates of Acinetobacter baumannii. Antimicrob Agents Chemother 54: 3569–3577. 20530228 10.1128/AAC.00057-10 PMC2934971

[22] Poirel L, Nordmann P (2006) Carbapenem resistance in Acinetobacter baumannii: mechanisms and epidemiology. Clin Microbiol Infect 12: 826–836. 16882287 10.1111/j.1469-0691.2006.01456.x

[23] Hu WS, Yao SM, Fung CP, Hsieh YP, Liu CP, et al (2007) An OXA-66/OXA-51-like carbapenemase and possibly an efflux pump are associated with resistance to imipenem in Acinetobacter baumannii. Antimicrob Agents Chemother 51: 3844–3852. 17724156 10.1128/AAC.01512-06 PMC2151406

[24] Corvec S, Caroff N, Espaze E, Giraudeau C, Drugeon H, et al (2003) AmpC cephalosporinase hyperproduction in Acinetobacter baumannii clinical strains. J Antimicrob Chemother 52: 629–635. 12951337 10.1093/jac/dkg407

[25] Turton JF, Ward ME, Woodford N, Kaufmann ME, Pike R, et al (2006) The role of ISAba1 in expression of OXA carbapenemase genes in Acinetobacter baumannii. FEMS Microbiol Lett 258: 72–77. 16630258 10.1111/j.1574-6968.2006.00195.x

[26] Segal H, Nelson EC, Elisha BG (2004) Genetic environment and transcription of ampC in an Acinetobacter baumannii clinical isolate. Antimicrob Agents Chemother 48: 612–614. 14742218 10.1128/AAC.48.2.612-614.2004 PMC321557

[27] Segal H, Garny S, Elisha BG (2005) Is IS(ABA-1) customized for Acinetobacter? FEMS Microbiol Lett 243: 425–429. 15686845 10.1016/j.femsle.2005.01.005

[28] Segal H, Jacobson RK, Garny S, Bamford CM, Elisha BG (2007) Extended −10 promoter in ISAba-1 upstream of blaOXA-23 from Acinetobacter baumannii. Antimicrob Agents Chemother 51: 3040–3041. 17548500 10.1128/AAC.00594-07 PMC1932550

[29] Zhou H, Pi BR, Yang Q, Yu YS, Chen YG, et al (2007) Dissemination of imipenem-resistant Acinetobacter baumannii strains carrying the ISAba1 blaOXA-23 genes in a Chinese hospital. J Med Microbiol 56: 1076–1080. 17644715 10.1099/jmm.0.47206-0

[30] Lee Y, Kim CK, Lee H, Jeong SH, Yong D, et al (2011) A novel insertion sequence, ISAba10, inserted into ISAba1 adjacent to the bla(OXA-23) gene and disrupting the outer membrane protein gene carO in Acinetobacter baumannii. Antimicrob Agents Chemother 55: 361–363. 20937784 10.1128/AAC.01672-09 PMC3019662

[31] Chen TL, Wu RC, Shaio MF, Fung CP, Cho WL (2008) Acquisition of a plasmid-borne blaOXA-58 gene with an upstream IS1008 insertion conferring a high level of carbapenem resistance to Acinetobacter baumannii. Antimicrob Agents Chemother 52: 2573–2580. 18443121 10.1128/AAC.00393-08 PMC2443897

[32] Poirel L, Figueiredo S, Cattoir V, Carattoli A, Nordmann P (2008) Acinetobacter radioresistens as a silent source of carbapenem resistance for Acinetobacter spp. Antimicrob Agents Chemother 52: 1252–1256. 18195058 10.1128/AAC.01304-07 PMC2292503

[33] Poirel L, Nordmann P (2006) Genetic structures at the origin of acquisition and expression of the carbapenem-hydrolyzing oxacillinase gene blaOXA-58 in Acinetobacter baumannii. Antimicrob Agents Chemother 50: 1442–1448. 16569863 10.1128/AAC.50.4.1442-1448.2006 PMC1426978

[34] Mussi MA, Limansky AS, Viale AM (2005) Acquisition of resistance to carbapenems in multidrug-resistant clinical strains of Acinetobacter baumannii: natural insertional inactivation of a gene encoding a member of a novel family of beta-barrel outer membrane proteins. Antimicrob Agents Chemother 49: 1432–1440. 15793123 10.1128/AAC.49.4.1432-1440.2005 PMC1068641

[35] Syn CK, Swarup S (2000) A scalable protocol for the isolation of large-sized genomic DNA within an hour from several bacteria. Anal Biochem 278: 86–90. 10640359 10.1006/abio.1999.4410

[36] Aranda J, Poza M, Pardo BG, Rumbo S, Rumbo C, et al (2010) A rapid and simple method for constructing stable mutants of Acinetobacter baumannii. BMC Microbiol 10: 279. 21062436 10.1186/1471-2180-10-279 PMC2993698

[37] Dorsey CW, Tomaras AP, Actis LA (2002) Genetic and phenotypic analysis of Acinetobacter baumannii insertion derivatives generated with a transposome system. Appl Environ Microbiol 68: 6353–6360. 12450860 10.1128/AEM.68.12.6353-6360.2002 PMC134429

[38] Zheng L, Baumann U, Reymond JL (2004) An efficient one-step site-directed and site-saturation mutagenesis protocol. Nucleic Acids Res 32: e115. 15304544 10.1093/nar/gnh110 PMC514394

[39] Seifert H, Dolzani L, Bressan R, van der Reijden T, van Strijen B, et al (2005) Standardization and interlaboratory reproducibility assessment of pulsed-field gel electrophoresis-generated fingerprints of Acinetobacter baumannii. J Clin Microbiol 43: 4328–4335. 16145073 10.1128/JCM.43.9.4328-4335.2005 PMC1234071

[40] Morita Y, Narita S, Tomida J, Tokuda H, Kawamura Y (2010) Application of an inducible system to engineer unmarked conditional mutants of essential genes of Pseudomonas aeruginosa. J Microbiol Methods 82: 205–213. 20538017 10.1016/j.mimet.2010.06.001

[41] Peleg AY, Adams J, Paterson DL (2007) Tigecycline Efflux as a Mechanism for Nonsusceptibility in Acinetobacter baumannii. Antimicrob Agents Chemother 51: 2065–2069. 17420217 10.1128/AAC.01198-06 PMC1891386

[42] Hornsey M, Ellington MJ, Doumith M, Thomas CP, Gordon NC, et al (2010) AdeABC-mediated efflux and tigecycline MICs for epidemic clones of Acinetobacter baumannii. J Antimicrob Chemother 65: 1589–1593. 20554571 10.1093/jac/dkq218

[43] Magnet S, Courvalin P, Lambert T (2001) Resistance-nodulation-cell division-type efflux pump involved in aminoglycoside resistance in Acinetobacter baumannii strain BM4454. Antimicrob Agents Chemother 45: 3375–3380. 11709311 10.1128/AAC.45.12.3375-3380.2001 PMC90840

[44] Coyne S, Guigon G, Courvalin P, Perichon B (2010) Screening and quantification of the expression of antibiotic resistance genes in Acinetobacter baumannii with a microarray. Antimicrob Agents Chemother 54: 333–340. 19884373 10.1128/AAC.01037-09 PMC2798560

[45] Park JY, Kim S, Kim SM, Cha SH, Lim SK, et al (2011) Complete genome sequence of multidrug-resistant Acinetobacter baumannii strain 1656-2, which forms sturdy biofilm. J Bacteriol 193: 6393–6394. 22038960 10.1128/JB.06109-11 PMC3209198

[46] Adams MD, Goglin K, Molyneaux N, Hujer KM, Lavender H, et al (2008) Comparative genome sequence analysis of multidrug-resistant Acinetobacter baumannii. J Bacteriol 190: 8053–8064. 18931120 10.1128/JB.00834-08 PMC2593238

[47] Vallenet D, Nordmann P, Barbe V, Poirel L, Mangenot S, et al (2008) Comparative analysis of Acinetobacters: three genomes for three lifestyles. PLoS One 3: e1805. 18350144 10.1371/journal.pone.0001805 PMC2265553

[48] Murat D, Bance P, Callebaut I, Dassa E (2006) ATP hydrolysis is essential for the function of the Uup ATP-binding cassette ATPase in precise excision of transposons. J Biol Chem 281: 6850–6859. 16407313 10.1074/jbc.M509926200

